# Incidence and Risk Factors of Colistin-Induced Nephrotoxicity Associated with The International Consensus Guidelines for the Optimal Use of the Polymyxins: A Retrospective Study in a Tertiary Care Hospital, Saudi Arabia

**DOI:** 10.3390/antibiotics11111569

**Published:** 2022-11-07

**Authors:** Fawaz M. Alotaibi, Bashayer M. Alshehail, Zainab A. H. Al Jamea, Royes Joseph, Amal H. Alanazi, Najla A. Alhamed, Reyouf S. Alqarni

**Affiliations:** 1 Pharmacy Practice Department, College of Clinical Pharmacy, Imam Abdulrahman Bin Faisal University, Dammam 31441, Saudi Arabia; 2 Pharmaceutical Care Department, King Fahd Hospital of The University/Imam Abdulrahman Bin Faisal University, Dammam 31441, Saudi Arabia; 3 College of Clinical Pharmacy, Imam Abdulrahman Bin Faisal University, Dammam 31441, Saudi Arabia

**Keywords:** nephrotoxicity, antibacterial, adverse drug reactions, colistin, retrospective study, pharmacoepidemiology

## Abstract

**Background:** Colistin is an effective therapy against multidrug-resistant gram-negative bacteria. However, nephrotoxicity is a major issue with its use. **Objective:** We aimed to evaluate the incidence and the potential risk factors of nephrotoxicity in colistin-treated patients. **Methods:** A retrospective cohort study was conducted. All adult patients aged 18 years and older who received colistin for ≥72 h were included in the study, while end-stage kidney disease patients requiring dialysis or had renal transplants were excluded. The incidence and severity of acute kidney injury (AKI) were assessed based on the Kidney Disease Improving Global Outcomes (KDIGO). **Result:** Out of 128 patients who received colistin, 51.56% of them have experienced AKI. The incidence was increased among oldest patients (above 80) and those who did not receive the appropriate dose (*p*-value = 0.0003). In addition, the median time until the AKI occurred was 10 days after receiving the colistin treatment. Rates of AKI in patients with previous AKI (71.7%) were three times higher than patients who did not previously experience AKI (HR = 2.97, 95% CI [1.8–4.8]). **Conclusions:** Nephrotoxicity is a significant issue among patients who receive colistin in the hospital, especially among older patients and those who did not receive the appropriate dose. As a result, healthcare providers should play a major role in colistin dosing, especially among the older adult population.

## 1. Introduction

Steadily increasing antibiotic resistance is a universal public health threat [1,2,3]. Of particular concern is the alarming increase in the prevalence of multi-drug and extensively-drug resistant gram-negative organisms among nosocomial-acquired infections. The gram-negative microorganisms of major concern are *Acinetobacter baumannii*, *Pseudomonas aeruginosa*, and *Klebsiella pneumoniae* [2,3,4,5,6,7]. The wide spread of MDR limits the choices of effective antibiotics against these organisms, which increases the complexity of the treatment plan [2,8]. Consequently, colistin has been re-adopted in the early 2000s as a core salvage therapy to tackle the deleterious influence of these infections [2,4,6,8,9,10].

Colistin (polymyxin E) is a naturally isolated antibiotic that was discovered in 1950. However, the role of colistin as a therapeutic option decreased in the 1970s as it was associated with nephrotoxicity, and antibiotic agents with improved safety profiles have become readily available [5,7,8,11,12,13,14]. Several studies have shown that colistin effectively treats multidrug-resistant gram-negative organisms. However, the cure and mortality rates have been reported as 75% and 60%, respectively [1,7,15,16].

Colistin-induced nephrotoxicity remains the major dose-limiting adverse event associated with its use [14] and although its efficacy has been well established for decades, the incidence rate of AKI, the time-to-AKI, and the potential predisposing factors have not been well-defined yet [9]. The incidence of colistin-induced AKI was reported to be between 20–60% [4,14,17]. This heterogeneity between studies could be attributed to using various definitions of AKI, the inclusion of different patient populations, and the use of a wide range of dosing regimens of colistin [14,17,18,19]. The time-to-incidence of acute kidney injury in patients treated with colistin has been shown to range between 5–12 days [4,5,6,9,19]; nephrotoxicity is usually reversible upon dose modification or treatment cessation [5,6]. Various colistin-associated AKI risk factors have been reported, including the prolonged duration of colistin therapy [14,17], coadministration of nephrotoxic agents [4,14,17,20,21,22], advanced age [4,6,17,23,24], higher weight independent of the dose [17], underlying disease severity [4], baseline renal function [5,6], hypoalbuminemia [4,17], high Charlson Comorbidity index score (CCI score) [4,25], and chronic comorbidities such as diabetes mellitus, chronic obstructive pulmonary disease, chronic liver disease, and hypertension [5,17,22].

Since the launch of the new International Consensus Guidelines for Optimal Use of Polymyxins in early 2019, a limited number of studies has been published to assess the incidence rate of AKI and the predisposing risk factors in light of these newly recommended doses [14]. Hence, an expanded view on these factors is of particular importance. Moreover, positive health-related outcomes might be achieved once the effect of the potentially modifiable predisposing factors for AKI in colistin-treated patients is mitigated. Thus, well-defined risk factors are warranted.

As a great paucity exists in the literature published in this area on global and national levels, this study was conducted to evaluate the incidence and potential predisposing risk factors for acute kidney injury among patients treated with colistin, considering the newly recommended doses.

## 2. Methods

**Study Design and Setting:** This was a retrospective study of patients treated with colistin at a tertiary care hospital, King Fahad Hospital of the University (KFHU) in the Eastern Province, Saudi Arabia. KFHU is a teaching and referral hospital with a 550 bed capacity.

**Study Participants:** Patients were included if they were 18 years of age and older, received colistin for ≥72 h for a documented resistant gram-negative bacterial infection, and had daily serum creatinine monitoring. In addition, patients with end-stage renal disease (ESRD), who required any form of renal replacement therapy (RRT) at the time of colistin initiation, or renal transplant patients were excluded. For patients who received more than one course of colistin, only the first course was included in the analysis. The informatics department generated a list of all patients who received colistin between January 2019 and June 2021.

**Study Outcomes:** The primary outcome of the study is the incidence of AKI among colistin-treated patients and the predisposing risk factors were considered as secondary outcomes. The incidence of AKI was assessed based on the KDIGO guideline definition. The KDIGO Clinical Practice Guidelines for Acute Kidney Injury define AKI as an increase in serum creatinine (SCr) by ≥0.3 mg/dL (≥26.5 μmol/L) within 48 h, or an increase in SCr ≥1.5 times baseline, which is known or presumed to have occurred within the prior 7 days, or a urine volume <0.5 mL/kg/h for 6 h [24]. Due to the retrospective nature of the present study, the urine volume was not assessed as the urinary output-related information might potentially be unavailable.

**Data Collection:** A standardized data collection form that is constructed of three main domains; patient-related variables, admission-related variables, and therapeutic management-related variables was utilized for collecting the data from patients’ medical records using the hospital’s computerized system (QuadraMed^®^Plano, Plano, TX, USA). All the data we have received from the informatics department were deidentified data to protect patient confidentiality according to IAU-IRB’s policy.

The first domain was designed to collect patient-related information that includes patient demographics (i.e., age, gender, weight, and BMI), comorbidities (i.e., diabetes mellitus, hypertension, heart failure, chronic kidney disease, cancer, and others), Charlson comorbidity index score, and renal function (i.e., baseline renal function “SCr and estimated creatinine clearance (eCrCl), which was calculated based on Cockcroft and Gault formula”, previous acute kidney injury, AKI after colistin therapy, degree of severity of AKI, and reversibility of AKI).

The second domain contains admission-related information such as the level of care (i.e., critical versus non-critical), number of days of hospital stay, and recent hospital admissions within the previous 90 days.

Lastly, the therapeutic management-related information domain was incorporated three major parts: colistin-related information (i.e., dose of intravenous colistin per day, total daily dose if more than one route of administration was utilized, total cumulative dose of colistin, duration of colistin therapy in days, and appropriateness of the dose), concomitant therapies (i.e., antimicrobials and non-antimicrobial therapy), and microbiological data (i.e., site of infection and causative micro-organism). Appropriateness of each medication/dose was assessed by a team of clinical pharmacists specialized in infectious diseases and critical care patients.

**Statistical Analysis:** Data were summarized through mean and standard deviation (SD) for continuous variables and frequency and percentage for categorical variables. The time to the incidence of AKI was calculated as the time difference in days between administration of colistin and the incidence of AKI. The median incidence time and incidence rate were estimated using the Kaplan–Meier curve. The Cox proportional hazards model was used to examine the association between the incidence time and the set of predictor variables. The Hazard Ratio (HR) was measured to identify risk factors for the occurrence of the incidence of AKI. We estimated HRs with a 95% Confidence Interval (CI) in adjusted and unadjusted regression models. Variables found significant in unadjusted models or with theoretical importance were included in the adjusted model, where the interaction effect was also examined. Missing data were assumed missing at random for the regression models. Multiple imputation using chained equations was performed to create 60 complete datasets with 10 initial burn-in iterations [26]. The imputed variables (% of missing data) were BMI (21.9%), CCI (6.3%), DM (10.9%), HTN (7%), eCrCl (5.5%), previous AKI (1.6%), previous hospitalization (1.6%), inotropic support (9.4%), CRRT (9.4%), appropriateness of the dose (6.3%), loop diuretics (0.8%), NSAIDs (0.8%), A Baumannii (0.8%), and K Pneumoniae (0.8%). The imputation models included all covariates in the estimation model, AKI event indicator, and the corresponding Nelson–Aalen estimator of the cumulative hazard (White and Royston, 2009). Results with *p*-values less than 0.05 were considered statistically significant. All analyses were performed using Stata/SE 16.1 [27].

**Ethical considerations:** The study received approval from the Deanship of Scientific Research at Imam Abdulrahman bin Faisal University, Dammam, Saudi Arabia (IRB number: IRB-UGS-2021-5-085). Informed consent was not obtained from the participants given the retrospective nature of the study.

## 3. Results

### 3.1. Baseline Characteristics of the Study Population

One hundred and twenty-eight patients met the inclusion criteria, and their baseline characteristics are presented in Table 1. Among the included participants, 45 (35.2%) were older adult (older than 65 years), 81 (63.3%) were males, 65 (50.8%) had a BMI > 25 kg/m^2^, 69 (53.9%) with DM, 69 (53.9%) with HTN, and 71 (55.8%) with moderate-severe CCI. Further, 46 patients (35.9%) had a previous history of AKI, 42 (32.8%) had a previous hospital admission within the past 90 days, 56 (43.8%) were on inotropic support, and 15 (11.7%) received CRRT. Importantly, 52 (40.6%) had inappropriate colistin doses. Vancomycin, Meropenem, and NSAIDs were co-administered with colistin on 33 (26.0%), 79 (62.2%), and 29 (22.7%) patients, respectively.

### 3.2. Incidence of AKI

**Figure 1:**
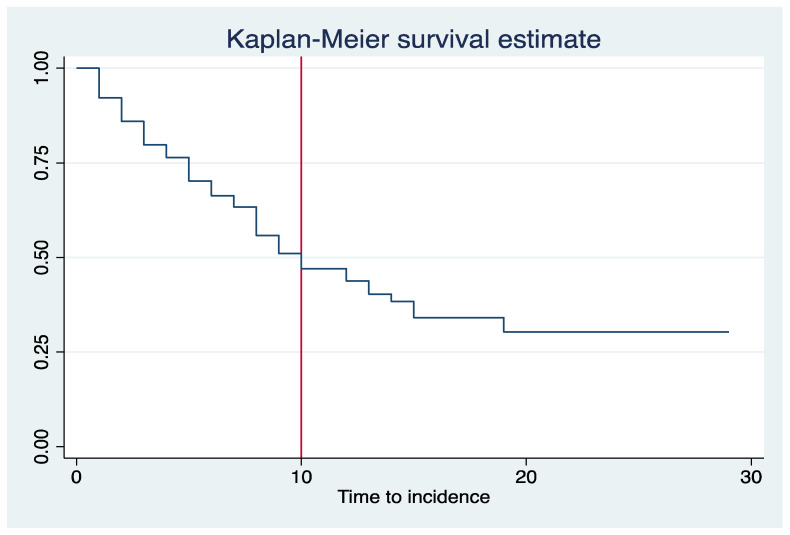
Kaplan–Meier curve for the incidence of AKI among colistin-treated patients.

Sixty-six (51.6%) patients had AKI during the hospitalization following colistin administration with an incidence rate of 6.41 per 100 person-days. The median incidence time was 10 days (95% CI: 8 to 14 days) (Figure 1). Regarding the severity of AKI, 14 (21.2%), 19 (28.8%), and 33 (50.0%) were on stage 1, 2, and 3, respectively. In addition, more than half of our population had experienced reversible AKI, which occurred in 37 (56.1%) patients.

### 3.3. Predictors of AKI

The number of incident cases in each level of prognostic factors are presented in Table 1. The unadjusted regression model showed that older adults, DM, HTN, higher CCI score, history of AKI, previous hospitalization in the past six months, inotropic support, CRRT, and patients on loop diuretics or NSAIDs were significantly associated with the higher incidence of AKI. Importantly, patients with an appropriate dose of colistin had a significantly reduced risk of AKI [unadjusted HR: 0.44 (0.27–0.73)]. Table 2 presents the adjusted HR with (95% CI) for the occurrence of AKI. The model showed a statistically significant interaction effect between age and CCI, which indicates differential effect of CCI on risk of AKI within age groups. Further, age, eCrCl, history of AKI incidence, Inotropic support, CRRT, and NSAIDs were significantly associated with the increased risk of AKI. Although the rate of AKI decreased when the patients received an appropriate colistin dose, the association was not statistically significant.

## 4. Discussion

Colistin dosing was standardized by the International Consensus Guidelines for the Optimal Use of the Polymyxins and endorsed by many infectious disease societies in 2019. The updated guideline is recommending higher doses than what was used previously, as lower dosses are associated with treatment failure and resistance. The main concern with these doses is the safety profile in terms of nephrotoxicity. The primary objective of this retrospective study was to evaluate the incidence of AKI and its predisposing risk factors while using the updated recommendations. Based on our best knowledge, this is the first study in the region after the 2019 guidelines has been released. In this study, the incidence of colistin-induced nephrotoxicity was 51.6% among patients treated against multi-drug and extensively-drug resistant gram-negative organisms, which is approximately similar to previously reported studies (21–75%) [6,28,29]. While the consensus guidelines for the optimal use of polymyxins estimate nephrotoxicity rates of 20–50%, our findings suggested differently, and that could be related to differences and variability in AKI definition, patient population, and the presence of potential confounders such as concomitant use of other nephrotoxic agents [6,28,29].

Although this study was performed after the implementation of the updated guidelines, in our practice, half of the patients received inappropriate doses, and they experienced a higher incidence of AKI than those who received the updated dosing recommendation. This might be due to the variability in dose adjustments based on calculated creatinine clearance. Accordingly, this has highlighted the importance of developing an institution-based guild to aid healthcare providers in adjusting the doses and choosing the right dose and frequency.

A meta-analysis of randomized controlled trials compared the nephrotoxicity of colistin versus beta lactam-based regimens and showed that colistin had increased this risk by 140% [30]. However, the efficacy of colistin toward MDR gram-negative organisms outweighs the risk in some patients, and it is therefore used as an effective last line option. Our study confirmed that advanced age is a significant risk factor for AKI [6,8,15]. In addition, higher creatinine levels were observed in this age group as well. Previous research has highlighted the importance of monitoring renal function in older adult patients [29]. Moreover, in our population, the incidence of nephrotoxicity was greatly influenced by the need for ICU admission, CRRT, and the use of inotropes. The use of loop diuretics and NSAIDs also increase the risk of AKI, which is expected as those medications directly affect the kidney. Similar trends and risk factors have been observed in previously reported studies that were conducted in 2020 and 2021 [28,30]. Although our study had similar findings, we had the advantage to be able to use CCI to test the association with the severity of AKI. Our findings suggest that patients with high CCI score had a 50% increased risk of AKI incidence compared to other patients with lower CCI score. Thus, more emphasis should be put on patients with comorbidities and polypharmacy to avoid such complications.

There are several limitations related to our study. The first limitation is that our study is a retrospective and single-centre study, which limits the generalizability of the study results. The second limitation is the low sample size, which is insufficient to identify possible significant associations. However, there are strengths in our study, which is the first study conducted in the Eastern Province of KSA related to colistin-induced nephrotoxicity after the implementation of the consensus guidelines for the optimal use of polymyxins in 2019. In addition, our study considers one of the views that uses the KDIGO definition instead of RIFLE to determine colistin-induced nephrotoxicity. Such tools enhance the quality of our study by measuring the AKI correctly and more accurately compared to other available tools [31].

## 5. Conclusions

Colistin is one of the limited therapeutic options for the management of MDR gram-negative organisms in hospital settings. In this retrospective cohort study, nephrotoxicity (as defined by KIDGO criteria) occurred among 51.6% of treated patients. Nephrotoxicity is a prevalent issue among patients who received colistin treatment in our setting and has serious adverse effects. Colistin therapy should be accompanied by carefully monitored serum creatinine levels, modification of the dose according to renal function, and avoidance of co-administration of other nephrotoxic agents; this will decrease the potential of the nephrotoxic effect of this effective antibiotic. There are limited studies in the literature about colistin nephrotoxicity worldwide. Thus, more studies are required to support the significance of the incidence of nephrotoxicity and risk factors associated with colistin use, especially after the implementation of the new dosing regimens to provide insight on strategies that maximize target therapeutic outcomes and optimize patient safety.

## Figures and Tables

**Table 1:** Baseline characteristics of study patients and incidence of AKI.

Variables	Total n (%)	Incidence n (%)	Unadjusted HR * (95% CI)	*p*-Value **
**Age (in years)**				
24–45	36 (28.1%)	10 (27.8%)	ref.	
46–65	47 (36.7%)	26 (55.3%)	3.39 (1.62–7.12)	0.001
66–80	18 (14.1%)	10 (55.6%)	3.60 (1.48–8.77)	0.005
above 80	27 (21.1%)	20 (74.1%)	4.17 (1.93–9.01)	0.000
**Gender**				
F	47 (36.7%)	23 (48.9%)	ref.	
M	81 (63.3%)	43 (53.1%)	1.01 (0.61–1.68)	0.972
**BMI**				
<25	35 (27.3%)	18 (51.4%)	ref.	
25–29.9	36 (28.1%)	21 (58.3%)	1.28 (0.66–2.46)	0.467
30 or above	29 (22.7%)	18 (62.1%)	1.17 (0.6–2.28)	0.643
**DM**				
No	45 (35.2%)	15 (33.3%)	ref.	
Yes	69 (53.9%)	43 (62.3%)	2.51 (1.4–4.49)	0.002
**HTN**				
No	50 (39.1%)	20 (40%)	ref.	
Yes	69 (53.9%)	42 (60.9%)	2.0 (1.17–3.42)	0.012
**Charlson Comorbidity index (CCI)**				
mean (SD) ^1^	3 (3)	3 (2) vs. 4 (3)	1.11 (1.02–1.21)	0.011
No 0	21 (16.4%)	7 (33.3%)	ref.	
Mild 1–2	28 (21.9%)	13 (46.4%)	1.75 (0.73–4.21)	0.214
Moderate 3–4	31 (24.2%)	18 (58.1%)	2.13 (0.98–4.63)	0.055
Severe 5 or above	40 (31.3%)	25 (62.5%)	2.74 (1.31–5.71)	0.007
**eCrCl range**				
10–29	8 (6.3%)	3 (37.5%)		
30–49	19 (14.8%)	16 (84.2%)	3.9 (1.13–13.49)	0.032
50–59	12 (9.4%)	10 (83.3%)	3.39 (0.93–12.34)	0.064
60–79	11 (8.6%)	3 (27.3%)	1.05 (0.21–5.16)	0.950
≥80	71 (55.5%)	32 (45.1%)	1.25 (0.38–4.06)	0.715
**Previous AKI**				
No	80 (62.5%)	31 (38.8%)	ref.	
Yes	46 (35.9%)	33 (71.7%)	2.93 (1.78–4.8)	0.000
**Recent hospital admission within 90 days**				
No	84 (65.6%)	35 (41.7%)	ref.	
Yes	42 (32.8%)	29 (69%)	1.71 (1.04–2.79)	0.033
**Level of care**				
Critical	101 (79)	53 (52.48)	ref.	0.4839
Non-critical	25 (19)	12 (52.17)	0.619 (0.145–2.642)	
**Length of hospitalization** Mean (SD)	70 days (61)	80 (67) vs. 58 (53)	0.998 (0.995–1.00)	0.2005
**Duration of therapy with Colistin**				
Mean (SD)	12 (7.8)	14 (8) vs. 10 (8)	0.966 (0.940–0.993)	0.0144
**Inotropic Support**				
No	60 (46.9%)	25 (41.7%)	ref.	
Yes	56 (43.8%)	38 (67.9%)	1.82 (1.09–3.03)	0.022
**CRRT**				
No	101 (78.9%)	50 (49.5%)	ref.	
Yes	15 (11.7%)	13 (86.7%)	2.55 (1.33–4.91)	0.005
**Appropriateness of the dose**			
No	52 (40.6%)	34 (65.4%)	ref.	
Yes	68 (53.1%)	29 (42.6%)	0.44 (0.27–0.73)	0.001
**Loop diuretics**				
No	70 (54.7%)	32 (45.7%)	ref.	
Yes	57 (44.5%)	34 (59.6%)	1.82 (1.11–2.99)	0.018
**NSAIDs**				
No	98 (76.6%)	46 (46.9%)	ref.	
Yes	29 (22.7%)	20 (69%)	2.2 (1.3–3.75)	0.004
**Vancomycin**				
No	94 (74.0%)	48 (51.1%)	ref.	
Yes	33 (26.0%)	18 (54.6%)	1.03 (0.59–1.77)	0.928
**Meropenem**				
No	48 (37.8%)	29 (60.4%)	ref.	
Yes	79 (62.2%)	37 (46.8%)	0.63 (0.39–1.04)	0.071
**UTI Infection**				
No	68 (53.1%)	31 (45.6%)	ref.	
Yes	59 (46.1%)	35 (59.3%)	1.33 (0.82–2.17)	0.245
**Pnumonia Infection**				
No	34 (26.5)	17 (50)	Ref.	
Yes	93 (72.66)	49 (52.69)	0.928 (0.625–1.378)	0.7125
**Wound Infection**				
No	88 (68.75)	39 (44.32)	ref	
Yes	39 (30.47)	27 (70)	0.628 (0.425–0.929)	0.019
**Bacteremia**				
No	50 (39.37)	24 (48)	Ref	
Yes	77 (60.63)	42 (54.55)	0.971 (0.678–1.390)	0.8717
**A Baumannii**				
No	31 (24.2%)	13 (41.9%)	ref.	
Yes	96 (75%)	53 (55.2%)	1.0 (0.54–1.85)	0.989
**K Pneumoniae**				
No	59 (46.1%)	26 (44.1%)	ref.	
Yes	68 (53.1%)	40 (58.8%)	1.36 (0.83–2.23)	0.223
**Albumin level**				
mean (SD) ^1^	2.64 (0.6)	2.71 (0.6) vs. 2.57 (0.7)	0.87 (0.58–1.31)	0.516
**Number of agents**				
mean (SD) ^1^	9 (5)	9 (5) vs. 9 (5)	1.0 (0.95–1.04)	0.856

^1^ mean (SD) of variables for non-AKI vs AKI patients. * Unadjusted Hazard Ratio. ** *p*-Value considered significant at 0.05. ref = reference group.

**Table 2:** Predictors of occurrence of AKI—adjusted model.

Variables		Adjusted HR (95% CI) *	*p*-Value **
Age			
24–45			
46–65		7.72 (1.88–31.68)	0.005
66–80		1.47 (0.13–16.27)	0.751
above 80		7.01 (0.99–49.71)	0.051
Interaction between Age & CCI			
24–45#CCI		1.21 (0.71–2.08)	0.481
46–65#CCI		0.58 (0.4–0.85)	0.005
66–80#CCI		0.93 (0.61–1.41)	0.732
above 80#CCI		0.71 (0.54–0.94)	0.017
DM			
No			
Yes		1.82 (0.85–3.92)	0.124
HTN			
No			
Yes		1.4 (0.71–2.79)	0.334
eCrCl range			
10–29			
30–49		4.34 (1–18.82)	0.050
50–59		8.95 (1.74–46.03)	0.009
60–79		1.11 (0.18–6.88)	0.914
≥80		2.7 (0.48–15.32)	0.262
Previous AKI			
No			
Yes		2.13 (1.03–4.41)	0.041
Recent hospital admission within 90 days			
No			
Yes		1.24 (0.66–2.32)	0.496
Inotropic Support			
No			
Yes		2.02 (1.05–3.88)	0.036
CRRT			
No			
Yes		2.9 (1.25–6.73)	0.013
appropriateness of the dose			
No			
Yes		0.5 (0.16–1.55)	0.228
Loop diuretics			
No			
Yes		1.26 (0.69–2.28)	0.453
NSAIDs			
No			
Yes		2.57 (1.26–5.24)	0.009
Vancomycin			
No			
Yes		0.99 (0.41–2.39)	0.979
Meropenem			
No			
Yes		0.64 (0.32–1.27)	0.200

* Adjusted Hazard Ratio. ** *p*-Value considered significant at 0.05.

## Data Availability

All collected data for this study are published in this article.
